# New Insights into the Activities of D-Chiro-Inositol: A Narrative Review

**DOI:** 10.3390/biomedicines9101378

**Published:** 2021-10-02

**Authors:** Riccardo Gambioli, Mario Montanino Oliva, Maurizio Nordio, Alfonsina Chiefari, Giulia Puliani, Vittorio Unfer

**Affiliations:** 1 R&D Department, Lo.Li. Pharma, 00156 Rome, Italy; gambioli.riccardo@gmail.com; 2 The Experts Group on Inositol in Basic and Clinical Research (EGOI), 00161 Rome, Italy; mario.montanino@artemisia.it (M.M.O.); maurizionordio1@gmail.com (M.N.); 3 Department of Obstetrics and Gynecology, Santo Spirito Hospital, 00193 Rome, Italy; 4 Department of Experimental Medicine, Sapienza University, 00185 Rome, Italy; 5 Oncological Endocrinology Unit, IRCCS Regina Elena National Cancer Institute, 00144 Rome, Italy; alfonsina.chiefari@ifo.gov.it (A.C.); giulia.puliani@ifo.gov.it (G.P.); 6 System Biology Group Lab, 00161 Rome, Italy

**Keywords:** inositol, D-chiro-inositol, insulin, steroidogenesis, integrins, inflammation

## Abstract

D-chiro-inositol (DCI) is a natural compound detectable in cell membranes, which is highly conserved as a biological signaling molecule. In mammals, its function is primarily characterized in the intracellular transduction cascade of insulin. In particular, insulin signal promotes the release of pivotal DCI-containing molecules. In fact, impaired release of DCI is a common feature of insulin-resistant tissues, and insulin-sensitizing pharmaceuticals induce higher concentrations of free DCI. Moreover, it also plays important roles in several other processes. DCI is involved in the regulation of steroidogenesis, due to its regulatory effects on steroidogenic enzymes, including 17α-hydroxylase, 3β-hydroxysteroid dehydrogenase, and aromatase. Such regulation of various enzymes indicates a mechanism by which the body regulates different processes via a single molecule, depending on its concentration. DCI also reduces the expression of integrin β3, which is an adhesion molecule involved in embryo implantation and cellular phenomena such as survival, stemness, and invasiveness. In addition, DCI seems to have important anti-inflammatory activities, like its 3-O-methyl-ether, called pinitol. In vitro evidence demonstrates that treatment with both compounds induces a reduction in pro-inflammatory factors—such as Nf-κB—and cytokines—such as TNF-α. DCI then plays important roles in several fundamental processes in physiology. Therefore, research on such molecule is of primary importance.

## 1. Introduction

D-chiro-inositol (DCI) is the second most represented isomer of the inositol family in mammals [1]. Inositols are cyclo-hexane polyols, differing from each other depending on the orientation of the six hydroxyl groups. DCI plays pivotal roles in several physiological processes and can be either absorbed through the diet or derived from its most represented relative, myo-inositol (MI) [2]. In fact, a NADH/NADPH-dependent epimerase enzyme specifically converts MI to DCI at physiological pH, either on the membranes or in the cytosol [3]. Both isomers are involved in membrane plasticity, participating in signal transduction by modulating the response to endocrinological stimuli [1]. Ultimately, both MI and DCI are catabolized by Myo-Inositol Oxygenase (MIOX), producing D-glucuronate, that eventually joins the pentose-phosphates cycle as D-xylulose-5-phosphate [4].

Throughout the whole body, each tissue or cellular type displays a peculiar ratio of MI to DCI. Actually, high MI/DCI ratios are present in almost every tissue, except for those deputed to storage, which display higher contents of DCI at the expense of MI [5]. Both MI and DCI exist in the intracellular space in one of their phosphate forms, either as inositol-phosphates (IP), free molecules in water solution, or as phosphatidylinositol-phosphate (PIP), hydrophilic components of the phospholipid bilayer [2]. Particularly, PIP may take part in a structure generally referred to as glycosylphosphatidylinositol anchor (GPI anchor). Indeed, through the addition of a glycan, generally composed of an amino-sugar and several saccharides, and a phosphoethanolamine linker, PIP connects to the C-terminal of a protein, resulting in proteins that are GPI-anchored to cell membranes. Noteworthy, GPI-anchored proteins represent a mechanism of primary importance, as defects in GPI anchor biosynthesis are lethal during embryo development in mammals [6].

Under unstimulated conditions, MI and DCI mainly exist as phosphatidylinositol-4,5-bisphosphate (PIP2), either unconjugated or associated with glycans. Following extracellular signals, PIP2 can undergo several modifications [7]. On the one hand, a phosphorylation of unconjugated inositol phosphates by Phosphatidylinositol-3-Kinase (PI3K) can produce phosphatidylinositol-3,4,5-trisphosphate (PIP3). On the other hand, Phospholipase C (PLC) can catalyze the release of inositol from cell membranes, producing inositol-1,4,5-trisphosphates (IP3) from unconjugated PIP2 [8]. Noteworthy, not only IP3 can be released from the membranes, but also inositol phosphoglycans (IPGs).

In all the processes involving inositol signaling, a difference between MI and DCI is not always clear. Nevertheless, MI content is lower in storage tissues as fat, muscle, and liver, while higher contents are found the other tissues [2]. This evidence, the available data on the mechanisms involving specifically DCI, and the data from clinical trials allow us to develop theories about molecular differences. This paper aims at evaluating those data, focusing on the actual and plausible roles played by DCI.

## 2. Insulin

Insulin is a well-known hormone produced by pancreatic β-cells, whose principal role is to promote cellular absorption of glucose. Insulin receptor is a tyrosine kinase transmembrane receptor existing as a dimer. Once the ligand binds, the receptor self-phosphorylates in the cytoplasmatic portion, allowing recognition by its interactors. Among these, Insulin Receptor Substrate 1 (IRS-1) and 2 (IRS-2) were demonstrated to interact with the inositol signaling pathway [8]. Particularly, both IRS-1 and IRS-2 interact with the p85 subunit of PI3K, whose role is to regulate the activity of the catalytic subunit p110, especially the isoforms p110α and p110β. Activated IRSs promote the phosphorylation of p85, reducing its inhibition of the coupled p110, and thus the insulin stimulus results in enhanced PI3K activity [9]. Interestingly, in physiology, the two p110 isoforms seem to have different downstream effects, especially on the proto-oncogenic protein Akt [10].

Therefore, the insulin stimulus promotes the formation of PIP3 via PI3K, leading to downstream signal transduction. On the other hand, via direct interaction [11], insulin induces an about three-fold higher activity of PLC-γ1, thus promoting the release of IP3 from the membranes to the cytosol. However, this generates a slight and transient depletion in PIP2, temporarily removing substrates for other processes such as the formation of GPI anchors [12].

**Figure 1:**
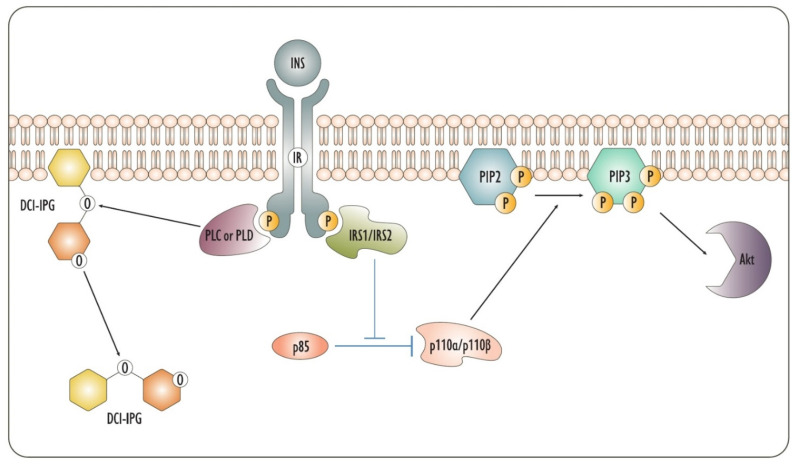
The figure depicts the insulin pathways based on inositol. On the one hand, IRS1 and IRS2 remove the inhibition of p85 on p110, thus promoting the conversion of PIP2 to PIP3, leading to the activation of downstream effectors such as Akt. On the other hand, an enzyme of the phosphodiesterase family, whose identity is, to date, debated and which could be PLC or PLD, cleaves DCI-IPGs from the membrane, allowing downstream transmission of the signal. DCI-IPG: DCI-based inositol phosphoglycans; INS: Insulin; IRS1/IRS2: Insulin Receptor Substrate 1/Insulin Receptor Substrate 2; PIP2: phosphatidylinositol-4,5-bisphosphate; PIP3: phosphatidylinositol-3,4,5-trisphosphate; PLC: Phospholipase C; PLD: Phospholipase D.

In the insulin pathway, DCI is considered a key molecule in the signaling cascade (Figure 1). In fact, DCI-based IPGs (DCI-IPGs) participate as signaling molecules in signal transduction by the insulin receptor. Particularly, the action of insulin promotes the phospholipase-mediated release of a DCI-IPG mediator. This DCI-IPG is a pseudo-disaccharide composed of galactosamine and pinitol, which is the 3-O-methyl ether of DCI [13]. In addition to the cytoplasm, extracellular environments like serum show the presence of DCI-IPG, whose role as an insulin mediator and an insulin sensitizer is widely described in the literature [7,14,15,16,17,18]. Noteworthy, DCI-IPGs in the bloodstream derives from phospholipase-mediated cleavage and release of DCI-IPGs from the outer part of the membranes. To trigger this mechanism, phospholipase is expressed as a GPI-anchored protein on the external layer of cell membranes, where it allows the extracellular release of DCI-IPGs [6,19]. To date, conflicting evidence exists on the phosphodiesterase that catalyzes the release of DCI-IPGs from the membrane. Sleight et al. found that a heterodimer of G-proteins is involved in the release of IPGs, also highlighting its colocalization with PLCβ and PLCδ [20]. Despite this, there is lack of evidence on the existence of an IPG-specific PLC in mammals, while it has been detected in bacteria [21]. Another hypothesis concerns the activity of Phospholipase D (PLD), which catalyzes the cleavage of IPGs between phosphate and inositol, rather than between phosphate and glycerol, as PLC does [21]. This cleavage seems to be of primary importance in insulin downstream effects, specifically, in glucose uptake [22]. Further evidence from Bonilla et al. highlighted that bovine-derived PLD specifically cleaves DCI-IPGs, while PLC purified from bacteria cannot [23]. Moreover, protein-free IPGs usually undergo palmitoyl–acylation on carbon 2, which prevents the cleavage of PLC but not that of PLD [21]. Nevertheless, due to the existence of a bovine PLC that hydrolyzes IPGs, the possibility of a similar lipase cannot be ruled out in humans, making the issue still debated [6].

Larner et al. proposed that DCI-IPGs derive from the hydrolysis of phospholipids in the membrane, from IPGs linked to proteins, or from both [13]. DCI-IPGs are also characterized as promoters of Pyruvate Dehydrogenase activity via the activation of Pyruvate Dehydrogenase Phosphatase [13]. In addition, DCI-IPGs also activate Protein Phosphatase 2C (PP2C) [24], which represents an important effector that further allows PIP3 production, as PP2C directly activates PI3K [25]. These two pathways in turn lead to insulin sensitization and promote energetic metabolism in the cells.

In pancreatic environment, DCI-IPGs stimulate insulin secretion from pancreatic β-cells. In fact, high glucose levels in the bloodstream induce a systemic higher activity of PLC, promoting the release of DCI-IPGs [26]. Eventually, DCI-IPGs induce the secretion of insulin via the closure of ATP-sensitive potassium channels. In fact, DCI-IPG treatment fails to potentiate insulin secretion following the chemically induced closure of ATP-sensitive potassium channels. Noteworthy, PP2C is strictly required for the closure of ATP-sensitive potassium channels stimulated by DCI-IPGs and, thus, for insulin release from pancreatic β-cells [27]. DCI also prevents palmitate-induced insulin resistance in pancreatic α-cells, whose role is to secrete glucagon, which would promote the release of glucose in the bloodstream [28]. Thus, impaired DCI signal may also alter glucagon homeostasis, thus impairing the secretion of glucose. Therefore, DCI-IPGs play a pivotal role in maintaining glucose homeostasis in human organisms. Further confirmation of these facts derives from an in vitro study on the effect of insulin and glucose on inositol uptake. Indeed, the insulin stimulus promotes the upregulation of Sodium/Myo-Inositol Transporter 2 (SMIT2), which transports both MI and DCI, while DCI transport is competitively inhibited by small quantities of glucose [29].

As suggested by several clinical trials, the release of DCI-IPGs strongly relates to insulin sensitivity [17,18]. In fact, impaired release of DCI-IPGs from cell membranes characterizes insulin-resistant subjects, and DCI administration improves insulin sensitivity, reducing insulin levels [30,31]. Moreover, patients affected by diabetes mellitus show enhanced urinary excretion of DCI and impaired levels of circulating DCI, demonstrating the pivotal role of such molecule [32].

Other than in the response to insulin, DCI is involved in the maturation of adipocytes. In particular, DCI induces the activation of IRS without upregulating the expression of the insulin substrate. On the contrary, insulin induces both the expression and the phosphorylation of IRS, resulting in an unchanged ratio of activated IRS to total IRS [33]. As a consequence, DCI partially mimics the effect of insulin, augmenting the relative activation of IRS to a greater extent. In fact, given the ability of DCI to improve IRS phosphorylation grade without upregulating the gene, the stimulated cell will better respond to additional insulin stimulus. Therefore, insulin resistance and impaired release of DCI further reduce insulin sensitivity, in a pathological, positive feedback.

Additional demonstrations on the importance of DCI in insulin physiology derive from the mechanisms of action of insulin-sensitizing pharmaceuticals. In particular, metformin and pioglitazone, two well-known insulin sensitizers, exert their roles through mechanisms involving the improvement of DCI-IPG release [17,34]. Therefore, the improved signal of insulin mediated by DCI-IPGs represents an important part of these pharmaceuticals’ mechanisms. However, insulin does not transduce only via DCI, and in a similar way DCI do not participate only in insulin signaling.

## 3. Steroidogenesis

Other than in insulin signaling, DCI proved to be pivotal in several other endocrine processes. Intriguingly, DCI also participates in the pathways of gonadotropins. Particularly, as it emerges from a clinical point of view, DCI may act as a Luteinizing Hormone (LH) sensitizer, reducing endogenous LH synthesis and improving LH signaling [35,36,37]. This is probably due to the involvement of inositols and inositol-phosphates in LH signaling pathway. However, the LH receptor is a complex protein involving not only inositol but also several other downstream mediators and effectors [38].

**Figure 2:**
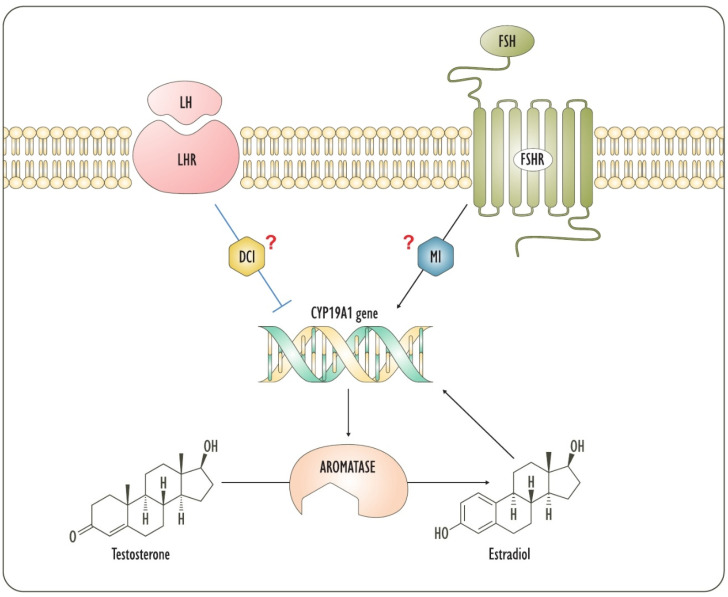
The figure depicts the regulation by LH and FSH of aromatase expression. Gonadotropins are involved in ovarian steroidogenesis through their intracellular mediators, which are strongly supposed to be inositols. Actually, LH signal involves principally theca cells, while FSH primarily acts on granulosa. DCI: D-chiro-inositol; FSH: Follicle-Stimulating Hormone; FSHR: Follicle-Stimulating Hormone Receptor; LH: Luteinizing Hormone; LHR: Luteinizing Hormone Receptor; MI: Myo-inositol.

Another evidence of the importance of DCI in hormonal regulation derives from its inhibition of the expression of aromatase [39,40]. Aromatase is an enzyme that catalyzes the aromatization of the A-ring of androgens to produce estrogens and it is the only enzyme that synthesizes estrogens. On the other hand, estradiol [41,42] and Follicle-Stimulating Hormone (FSH) [43,44] induce aromatase expression. Both estrogen and gonadotropin transduce through inositol phosphates [45,46,47,48]. Being DCI an inhibitor of aromatase expression, it is likely that estradiol and FSH block DCI signals in favor of MI, leading to the expression of aromatase. On the contrary, LH downregulates aromatase [43,49], as insulin does [40,50]. Remarkably, insulin-dependent inhibition of aromatase expression is mediated by DCI-IPGs [40]. Therefore, in the menstrual cycle, FSH induces the expression of the LH receptor and aromatase before ovulation. The latter is further supported by positive feedback from newly produced estrogens. A following peak of LH signal strongly inhibits aromatase [49,51], as confirmed by the decrease in the output of estrogens following the LH stimulus [52]. Based on the affinity with insulin effects, this LH-induced inhibition of aromatase expression is probably allowed by DCI-containing mediators (Figure 2).

In accordance with the interpretation of DCI as a promoter of higher androgen concentrations, in men DCI is detectable in higher content than in women, and males also display a lower MI/DCI ratio in the urine [53]. Though DCI seems to promote androgens synthesis, the mechanism involves the inhibition of aromatase, and so DCI actually inhibits androgen catabolism rather than promoting their biosynthesis. Intriguingly, low DCI levels do not trigger androgen accumulation. In fact, the LH receptor has a low affinity for proteins involved in the inositol pathway [38], and thus only extremely high levels of LH, as it occurs in men or in women during ovulation, inhibit aromatase [43]. On the contrary, the proteins associated with the insulin receptor have high affinity for PI3K [8]; therefore, even small DCI quantities allow sensitization to insulin. Therefore, insulin and LH likely share inositol signals, even if to different extents. Thus, distinct stimuli promote the release of different DCI quantities, which mediate different activities depending on the final DCI concentration.

Altered concentrations of DCI are involved in ovarian deregulated pathways in insulin-resistant subjects, who display a higher insulin content. In fact, the ovary never displays the insulin resistance phenomenon, therefore suffering from overburdening insulin stimuli in insulin-resistant patients [54]. As a consequence of this high insulin signaling, the membranes release high DCI-IPGs quantities, which result in ovarian testosterone accumulation [55].

**Figure 3:**
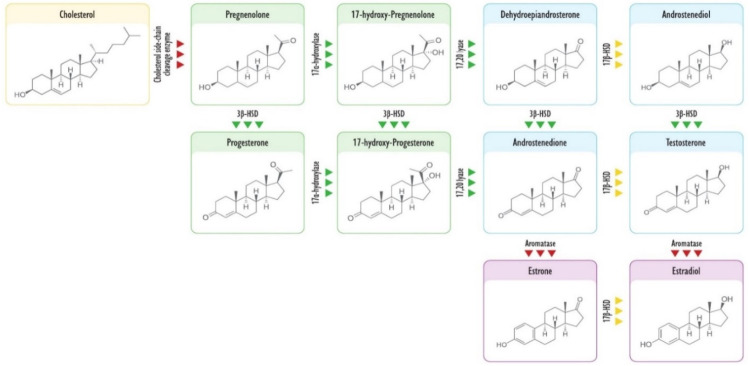
The figure reports the principal products of steroidogenesis and the enzymes involved. Yellow background depicts cholesterol; green background depicts progestogens; blue background depicts androgens; pink background depicts estrogens; green triangles indicate enzymes upregulated by DCI; yellow triangles indicate enzymes whose possible regulation by DCI is still unknown to date; red triangles indicate enzymes downregulated by DCI.

Intriguingly, both DCI and MI promote the activity of 3β-hydroxysteroid dehydrogenase (3β-HSD), which oxidizes the -OH group on the A-ring of progestogens and androgens. This is a regulation of primary importance during embryo development, particularly in the cytotrophoblast. In the latter, in fact, Nestler et al. demonstrated that both MI-IPGs and DCI-IPGs promote the production of progesterone in a concentration-dependent manner through a positive regulation on 3β-HSD [40]. Likewise, via PI3K, insulin improves the activity of 17α-hydroxylase, also known as 17,20 lyase, which catalyzes the production of androgens from progestogens [56]. In fact, diabetic patients with high insulin levels and impaired DCI signals show reduced conversion of progestogens into androgens [57]. This likely suggests that DCI induces the activity or the expression of 17α-hydroxylase, leading to a high conversion rate of progestogens to androgens. Nevertheless, DCI is also a transcriptional inhibitor of aromatase; thus, the insulin stimulus would result in an accumulation of androstenedione and testosterone at the expense of progestogens, dehydroepiandrosterone, androstenediol, and estrogens. Indeed, this imply that DCI may act as a promoter of androgens anabolism, also blocking their catabolism and thus avoiding the risks of anabolic steroids (Figure 3).

In physiological contexts, the insulin-dependent fine regulation of these enzymes would allow correct steroidogenesis to occur. However, in pathological clinical pictures such as diabetes and insulin resistance, an altered DCI signal would impair steroidogenesis, in addition to euglycemia. Particularly, women suffering from Poly-Cystic Ovary Syndrome (PCOS) usually display insulin resistance [5] and show increased DCI content in the ovary, coupled with a lack of DCI in non-germinal tissues [58]. Moreover, PCOS women display increased presence of steroidogenic enzymes in thecal and granulosa cells, including 17α-hydroxylase [59]. Therefore, treating PCOS women with insulin-sensitizing agents such as metformin reduces 17α-hydroxylase activity, allowing physiological steroidogenesis [60]. Concomitantly, the improved signals of insulin, that would lead to physiological signals via DCI, would also allow the recovery of the physiological expression and activity of aromatase and 3β-HSD. Therefore, DCI is nowadays considered an efficient insulin-sensitizing agent. However, at the ovarian level, high DCI quantities would exacerbate the impaired steroidogenesis, increasing the conversion of progestogens into androgens and impairing androgens catabolism. In fact, its administration in high content for a prolonged time seems to induce a PCO-like phenotype [61].

Intriguingly, the enhanced activity of 17α-hydroxylase in insulin-resistant women may represent a compensatory mechanism. In fact, in the case of altered insulin signaling, progesterone acts on the liver increasing blood glucose levels [62]. Therefore, the regulation by DCI of 17α-hydroxylase activity may derive from an adaptive mechanism to prevent the onset of a severer hyperglycemia. In this manner, the body would mitigate the effects of impaired insulin, inhibiting progesterone-induced hyperglycemia and thus avoiding more critical situations. However, the regulation by DCI of these enzymes leads to hyperandrogenism in pathological contexts involving impaired insulin signal [2].

## 4. Integrins

Other than the effects of DCI upon aromatase expression, Sacchi et al. [39] found further evidence of the effects of such a molecule on gene expression. Based on promising results from Lin et al. [63] involving treatment with pinitol, they demonstrated that DCI treatment also reduces the expression of integrin β3 in vitro. Integrins are transmembrane adhesion proteins, existing as heterodimers. Specifically, each mature integrin is composed of a specific α-chain and a specific β-chain. Interestingly, integrins not only provide adhesion to cells but also participate in cellular signaling pathways. In fact, liganded integrins transduce a signal of survival in epithelial cells through Focal Adhesion Kinase (FAK) and the proto-oncogene tyrosine protein kinase Src (c-Src). On the contrary, unliganded integrins do not provide a survival signal, inducing death in those cells that lose anchorage [64].

Physiologically, the β3 chain is detectable both in platelets, associated with the integrin αIIb or with the integrin αv, and in epithelial tissues, where it is commonly associated with the integrin αv. The ligands of αvβ3 include vitronectin, osteopontin, fibronectin, fibrinogen, and thyroxine [65]. In pathological contexts, β3 signal and mechanical anchorage is involved in several etiological processes, including cancer metastasis.

A low expression of integrin β3 is found in fat tissue, which is characterized by a high DCI content. However, β3 expression in fat tissue is related to body fat mass and insulin resistance. Thus, high body fat mass and insulin resistance induce high β3 expression [66]. Noteworthy, insulin-resistant tissues are characterized by a reduced DCI content [2]. Therefore, the fat mass of an insulin-resistant subject would display reduced levels of DCI, which in turn allow higher levels of β3 integrin. This could represent a cellular compensatory mechanism to restore insulin signal. In fact, β3 integrins interact with the insulin receptor, supporting its signal and likely amplifying the downstream cascade [64]. In this way, the correlation found in vitro by Sacchi et al. [39] between DCI and β3 integrins likely represents a physiological mechanisms of insulin sensitization.

Another physiological process involving integrin β3 as a factor of primary importance is embryo implantation. In fact, in uterine endometrium, integrin β3 is expressed from the early secretory phase to the menses and throughout the entire pregnancy [67]. On the other hand, also osteopontin [68] and vitronectin [69] are expressed during the same periods. Interestingly, also the embryo itself shows the expression of both integrin β3 and osteopontin [70]. Specifically, both osteopontin and integrin β3 are expressed on the surface of the trophoblast and at the implantation site [70]. Moreover, integrin β3 is necessary for the correct embryo implantation, as its inhibition leads to troublesome pregnancies or no pregnancy at all [71]. Intriguingly, the inhibition of integrin β3 leads to a higher relative abundance of natural killer cells and to a higher level of proinflammatory cytokines such as Interferon-γ, Tumor Necrosis Factor-α, and Interleukin-17 [71]. This is probably an eventual effect of failed implantation rather than a direct effect of a lack in integrin β3, as the body promotes the elimination of the trophoblast.

An indirect confirmation of the need for integrin β3 in physiological pregnancy derives from the analysis of endometrium samples from infertile women. Endometrium samples from women suffering from unexplained infertility [72] or recurrent pregnancy loss [73] were examined for their integrin β3 content. Researchers found that the endometria of both these populations of women are characterized by a low content of integrin β3. Further evidence is presented by the findings of Lessey et al. [74], who described the expression of integrin β3 in endometrium samples from healthy women and from women suffering from endometriosis, either fertile or infertile. They found that the normal expression of integrin β3 characterizes the endometrium of fertile women, regardless of their endometriosis condition, while infertile patients display reduced integrin β3 expression. This suggests that integrin β3 directly relates to fertility rather than to fertility-impairing pathologies.

Intriguingly, estrogens and progestogens seem to play important roles in the regulation of integrin β3. In particular, researchers found that estradiol reduces the expression of integrin β3, while the counteracting action of progesterone induces its expression in endometrial cells [75]. In this manner, progesterone improves the endometrial receptivity of embryos during the uterine secretory phase, preparing the milieu for implantation. Interestingly, progesterone induces the expression of integrin β3 via the induction of the expression of Heparin-Binding Epidermal-growth-factor-like-Growth-Factor (HBEGF) [76]. Noteworthy, HBEGF activity is mediated by PI3K, and thus the presence of PIP3 assists the eventual effects of progesterone on integrin β3 [77]. In this context, the diametrically opposite actions of insulin and progesterone emerge once again, as a high insulin signal reduces integrin β3 expression, while a high progesterone signal induces it.

## 5. Inflammation and Cancer

The inhibitory effects of pinitol and DCI upon integrin expression were further investigated in cancer, as these adhesion molecules play pivotal roles in cancer etiopathogenesis and progression. In fact, integrin β3 is considered a pro-tumorigenic integrin, as it relates to the metastatic and invasive processes, and its downregulation suppresses these phenomena [78]. Noteworthy, integrins can combine with membrane receptors with tyrosine kinase activity. The combination of an integrin and a receptor massively amplifies the signaling of both. In particular, integrin β3 showed combinatory activity with receptors of primary importance, including those for insulin, insulin-like growth factor 1 (IGF-1), Epidermal Growth Factor (EGF), and Vascular Endothelial Growth Factor (VEGF) [64]. Another important receptor that concomitantly supports and is supported by integrin β3 is Tyrosine Kinase receptor B (Trk-B) [79]. Trk-B is a membrane surface receptor that binds Brain-Derived Neurotrophic Factor (BDNF). This receptor coupled with integrin β3 promotes epithelial–mesenchymal transition and resistance to anoikis, i.e., detachment-induced death [80,81,82].

Another notable process involving integrin β3 in cancer is stemness maintenance and, thus, avoidance of differentiation. In fact, researchers found that integrin β3 is strictly required for the onset of some cancer types, such as acute myeloid leukemia, while its knockdown induces the differentiation of cancer cells [83]. This finding, in accordance with the high expression of integrin β3 in healthy and cancerous stem cells, underlines that integrin β3 may represent a hypothetical marker of stemness. Furthermore, integrin β3 counteracts the effects of chemotherapeutic inhibitors of the EGF receptor through stemness induction in cancer cells, allowing the establishment of resistance to these pharmaceuticals [84]. Of interest, the mechanisms underlying integrin-β3-mediated resistance to inhibitors of the EGF receptor seem to involve the activation of Nuclear Factor kappa-light-chain-enhancer of activated B cells (Nf-κB) [64].

Intriguingly, pinitol displayed anti-metastatic properties via the inhibition of the expression of integrin β3 and the reduction of the activity of c-Src and Nf-κB [63]. Particularly, pinitol seems to inhibit Nf-κB-induced genes, which include pro-inflammatory genes, such as cyclooxygenase-2 (COX2); genes related to proliferation, such as c-myc and cyclin D1; genes supporting survival, such as Bcl-2 and Bcl-xL; genes promoters of angiogenesis, such as VEGF; genes related to invasiveness, such as matrix metalloprotease-9 (MMP-9) [85]. Additionally, pinitol seems to reduce the synthesis of cytokines with pro-inflammatory activity, such as Tumor necrosis factor-α (TNF-α), and angiogenetic activity, such as Interleukin-8 [86]. It also modulates the immune response of T-helper cells, demonstrating a possible adjuvant effect in complex clinical pictures characterized by inflammation [87,88].

All these results concern pinitol, which is an ether of DCI, but most of these findings have not been confirmed for DCI yet. Nevertheless, DCI already proved to have similar and, in some cases, even better effects. In fact, firstly, DCI was shown to induce a greater reduction of the expression of integrin β3 than pinitol [39,63]. Secondly, DCI modulates the redox state and inflammation in adipocytes, downregulating TNF-α and Interleukin-6, which are modulator of the inflammatory response [89]. Moreover, DCI-IPGs demonstrated the ability to reduce the secretion of leptin, a pro-inflammatory factor, from adipocytes, even if to a lesser extent than MI-based IPGs [90].

Further evidence of the ability of DCI to prevent the onset of environments favoring malignancies derives from its effects on oxidative stress. In particular, DCI inhibits the expression of NADPH oxidase 4 (NOX4) and induces the activity Nuclear-factor-erythroid-2-Related Factor 2 (NRF2) [91]. NOX4 is a mitochondrial enzyme that produces free oxygen radicals, which increase oxidative stress and the inflammatory response of the cell [92]. Of interest, NRF2 is a key regulator in the homeostasis of oxidative stress and metabolism, which impacts on several other signaling cascades [93]. Therefore, in recent years, researchers focused their efforts on the search for pharmaceuticals that could enhance the effectiveness of NRF2 [93,94]. In this regard, DCI may likely represent a safe adjuvant treatment, reducing the inflammatory status and removing the integrin β3 stimulus to survival. Despite the encouraging in vitro evidence regarding both DCI [95,96] and pinitol [63,85,97,98,99] (Table 1), we should emphasize the lack of in vivo studies to date. If this evidence will be confirmed by appropriate in vivo data, cancer adjuvant treatment will represent an interesting field of application for a molecule of such potential.

## 6. Conclusions and Perspectives

In recent years, DCI has been widely used in clinical practice and investigated in clinical research. However, not all its physiological and therapeutical properties have been discovered yet, and further evidence is steadily emerging. Initially characterized as an insulin sensitizer and an insulin mimetic, today it is known that DCI plays pivotal roles also in the regulation of steroidogenesis and in other important processes, such as cell-to-cell adhesion and inflammation. Therefore, DCI is nowadays increasingly prescribed in clinical practice to treat a wide variety of diseases. In the future, research on the activity of DCI will undercover additional therapeutical properties of this natural and safe molecule, also gathering data on the molecular mechanisms underlying its therapeutic potential.

## Figures and Tables

**Table 1:** The table summarizes the in vitro evidence existing on the molecular regulation by DCI and Pinitol of genes relevant in cancer progression. c-Src: Proto-oncogene tyrosine protein kinase Src; COX2: cyclooxygenase-2; DCI: D-chiro-inositol; MMP-9: matrix metalloprotease-9; Nf-κB: nuclear factor kappa-light-chain-enhancer of activated B cells; NOX4: NADPH oxidase 4; NRF2: nuclear-factor-erythroid-2-related Factor 2; TNF-α: tumor necrosis factor-α; VEGF: vascular endothelial growth factor.

	Upregulated Genes	Downregulated Genes		Downregulated Genes
DCI	*NRF-2*	*Integrin β3*	Pinitol	*Integrin β3*
		*TNF-α*		*TNF-α*
		*IL-6*		*IL-8*
		*Leptin*		*NF-κB*
		*NOX4*		*c-Src*
				*c-myc*
				*COX-2*
				*Bcl-2*
				*Bcl-xL*
				*VEGF*
				*MMP-9*

## Data Availability

Not applicable.

## Abbreviations

3β-HSD: 3β-Hydroxysteroid dehydrogenase; BDNF: Brain-Derived Neurotrophic Factor; c-Src: Proto-oncogene tyrosine protein kinase Src; COX2: cyclooxygenase-2; DCI: D-chiro-inositol; DCI-IPGs: DCI-based IPGs; EGF: Epidermal Growth Factor; FAK: Focal Adhesion Kinase; FSH: Follicle-Stimulating Hormone; GPI-anchor: glycosylphosphatidylinositol anchor; HBEGF: Heparin-Binding Epidermal-growth-factor-like-Growth-Factor; IGF-1: insulin-like growth factor 1; IP: inositol phosphates; IPGs: inositol phospho-glycans; IP3: inositol-1,4,5-trisphosphates; IRS-1: Insulin Receptor Substrate 1; IRS-2: Insulin Receptor Substrate 2; LH: Luteinizing Hormone; MMP-9: matrix metalloprotease-9; MI: myo-inositol; MIOX: Myo-Inositol Oxygenase; Nf-κB: Nuclear factor kappa-light-chain-enhancer of activated B cells; NOX4: NADPH oxidase 4; NRF2: Nuclear-factor-erythroid-2-Related Factor 2; PCOS: Poly-Cystic Ovary Syndrome; PI3K: Phosphatidylinositol-3-Kinase; PIP: phosphatidylinositol phosphate; PIP2: phosphatidylinositol-4,5 bisphosphate; PIP3: phosphatidylinositol-3,4,5 trisphosphate; PLC: Phospholipase C; PLD: Phospholipase D; PP2C: Protein Phosphatase 2C; SMIT2: Sodium/Myo-Inositol Transporter 2; TNF-α: Tumor necrosis factor-α; Trk-B: Tyrosine Kinase receptor B; VEGF: Vascular Endothelial Growth Factor.
